# Reviews of Dental Literature

**Published:** 1901-12

**Authors:** 


### Reviews of Dental Literature.

---

A Contribution to the Sterilization of Dentine in Living and Dead Teeth.[1] By J. Choquet, D.E.D.P., F.M.P. (Paris).

Two years ago, at the Annual Meeting of the British Dental Association, held at Ipswich, I announced, when I presented the tubes of culture of micro-organisms obtained from dental decay, that before long I would prove that it is possible to stop the pathological process of this affection. It is these results I shall have the honor to present to you to-day.

Allow me first to remind you that the researches I have made on dental decay are quite different from those made till now by the many pioneers who have worked on the same subject. I have been the first to point out the truth of the theory advanced by Galippe and Miller that the destructive process of dental decay can continue under good fillings, and that notwithstanding all the care taken in the preparation of the cavities, notwithstanding the white and healthy aspect of the layer of dentine covering the pulp. It is thanks to the micro-organisms acting in time that I have succeeded in reproducing the disorders inherent to the affection upon a living animal, the sheep.

And to begin with, what must we understand by these words, sterilization of dentine?

Why, if we have taken every precaution in the preparation of the cavity, does this destructive process continue?

The reason for this is that, in the layer of dentine supporting the filling, and which, when examined microscopically, seems white, hard, and healthy, some microbic germs have still remained in it. These, on account of their anaërobic properties, finding in the contents of the dental canaliculi the necessary materials for their development, have continued to increase slowly, insensibly, at the same time occasioning the disorders every one knows. That is, if you like, a kind of the latent microbism of Verneuil.

Well, ideal antisepsis, as we understand it, means the whole of

---

[1] Read at the annual general meeting held in London, August, 1901.

the manipulations we have to effect in order to kill or at least diminish these micro-organisms before they have succeeded in reaching the pulp. One of our French colleagues, Dr. Mahé, has, I think, very well understood the question as we must understand it, He says,—

"The pulp has been accessible to the septic agent, and it is not, or it is no longer so to the antiseptic agent.

"The same layer of dentine which has been for the infection a ridiculously insufficient barrier, rises all powerful before the therapeutic agent."

In our opinion, it is impossible to place the question more clearly and more precisely, and that is the point on which we have to direct our attention more especially. That is, "allow the therapeutic agents to penetrate this barrier of dentine which is considered as impassable."

Without wishing to go into the details of all the processes successively used for the disinfection of dentine, whether we have to deal with the second or fourth degree of caries, let us remember, however, that long before microbes were spoken of, and consequently antisepsis, certain practitioners were accustomed, before filling, to paint over the cavities with a liquid of some kind. Why did they act so? It would be very difficult to answer this question. But these practitioners seemed to foresee the antagonistic action which microbes and antiseptics would have to play later. In any case, they thought that such a precaution, if it did not do any good, could not do any harm.

Then we had Pasteur's discoveries, afterwards those of Miller on ætio-bacteriology of dental decay. Immediately they thought of profiting by the antiseptic agents, and you know how small our therapeutic arsenal was at that time.

Certain practitioners thought of using carbolic acid, creosote, zinc chloride for the disinfection of their cavities, and they did that without thinking if any one of these liquids would penetrate the dentine (and this most often did not take place, as we shall see later), or supposing the contrary, if the liquid used did not go beyond the aim they had in view.

They thought only of one thing, to kill the microbes, without troubling themselves if at the same time they killed the cellular elements.

Gradually, as chemistry was developed, new antiseptics were added to those existing already. I will remind you, for the sake of argument only, that in America two quite opposite camps were formed about ten years ago for the disinfection, the sterilization of dead teeth. These two camps were formed of the partisans of the coagulant antiseptics and those for whom mere coagulants represented a perfect antiseptic. However, notwithstanding all the papers published, and the experiments made in the most conscientious manner, it has not yet been fully proved that there was really penetration and consequently absorption of the medicament destined to disinfect the dentine.

What is the reason for the bad results obtained? Simply that the medicaments used for the sterilization of the cavities *of living teeth* were used, so to speak, groping in the dark.[1]

This was, moreover and above all, because those who wished to obtain the sterilization of the cavities by washing them did not work in the laboratory, or, if they did so, they did not put into practice for the subject we are speaking of the rules inherent for the good preparation and conservation of histological preparations. The laboratory has been criticised, and very often it happens that serious people reason as follows: Laboratory? What is the good of that? Do you think that the experiments you make there can be of any use in daily practice?

Well, I wish to prove that the laboratory *is* useful in every-day practice, and that it is not only useful but absolutely indispensable to the persons who wish to work scientifically and practically, and that the results obtained *in anima vili* or *nobili,* according to the case, are only the results of the experiments made *in vitreo* in this same laboratory. Without going farther, why are the experiments in the sterilization of living dentine defective when carbolic acid

---

[1] Dr. Choquet does not add anything to the value of his paper, which is freely acknowledged, by belittling the work of others. The "two opposite camps in America," as he chooses to designate those who favored two quite different methods for the sterilization of the tubulated structure of dentine, performed very extensive laboratory work, and, so far as the writer was concerned, he demonstrated both macroscopically and microscopically the penetration of coagulants throughout the tubes. Dr. Choquet's method may be new, but if his dictum, that microbes penetrate the tubes beneath fillings, be true, then all fillings should prove a failure unless antiseptic processes are previously followed. This latter is quite a modern method, and yet fillings prior to the adoption of this preserved teeth from caries. His conclusions are not sustained by clinical experience.—Ed.

is employed, and when alcoholic antiseptics or the great majority of essential oils are used?

To this question we answer by saying that to wish to make one of the antiseptics above mentioned penetrate the dentinal canaliculi without having submitted the dentine previously to a special treatment is to wish to dissolve oil in water by simply floating it on the surface. It is to wish to clear a section of tissue obtained by freezing (that is to say, without any inclusion, and consequently aqueous, hydrated) by floating it directly in a bath of one of the clearing agents employed in histology. What will take place in that case? The section, however fine it may be, will remain in vain an indefinite time in that clearing agent: the water will never be driven out by the clearing agent.

On the contrary, if we dehydrate in a progressive manner this section of tissue by using alcohol of different strengths, and gradually stronger, we shall see it become clear and transparent when taken out of the liquid and put into a mixture of alcohol and clearing agent. The phenomenon observed for this section of tissue must be the same for the preparations to which we must resort, this barrier of dentine penetrable by microbic agents, but at the same time considered by Dr. Mahé as impossible for the therapeutic agents.

Before filling a cavity we must act in the following manner: (1) To clean the cavity as much as possible till we remove every portion of softened dentine; (2) dry the cavity and wash it with alcohol at seventy per cent.; (3) wash the same with absolute alcohol; (4) use a mixture of alcohol and toluol for washing the cavity; (5) dry and fill.

When I began the first experiments I could not, it is easily understood, do them in the mouth of my patients, so I was obliged to work in my laboratory. To make my experiments upon freshly extracted teeth did not please me on account of the density which exists between the different organs I had to work upon. So I thought it much better to act upon tissues approaching the nearest to the human dentine, and I began to work upon a hippopotamus tooth. I turned a few cylinders of ivory representing three or four centimetres in height and one in width, and with round burs I hollowed a fairly cylindrical cavity of two centimetres deep. I used one of these cylinders dry, and the other after having remained during an hour in a solution of chloride of sodium at one per cent.

Then I dried as much as I could the external portion with a linen rag and placed in the cavity a mixture of toluol and alcohol, to which I had added some methylene-blue. The same quantity of liquid was placed in the dry cylinder we shall call A and the moist one B. After five minutes in A I could see on the peripheral borders a slight blue tint, whilst nothing was apparently visible in B. Then I threw away the liquid of the two cylinders, washed them in water, and cut them longitudinally. As you can see, the cylinder A is almost wholly crossed by the staining agent, while in the cylinder B this staining is exclusively visible in the periphery of the cavity.

What are the conclusions we can deduce from those cylinders and teeth, from those dead and living tissues? The following: A layer of dentine which is not penetrated by certain liquids when it is moistened is entirely penetrated by the same agents if it has previously undergone certain preparations, as we have indicated before. Let us now consider how we can succeed in making an antiseptic penetrate the dentinal canaliculi, how we can prove the presence of this in the mass, and above all, how we can be assured that this antiseptic has performed its bactericidal properties. But before doing so allow me to make a little digression, and let me give to Cæsar what is Cæsar's. The first experiments I tried for the sterilization of dentine I performed in February, 1895, and when I published my first paper three months ago I thought I was the only one working in this way. (If I refer to my note-book I see I have treated since that time seven hundred and ninety-two teeth by the method indicated later, and I have not had any failures.) I was rather disappointed a few weeks ago, when making some bibliographical researches, to find in the copy for May, 1898, of the American journal, *Items of Interest,* an interview with the president of the Microscopical Section, my excellent colleague, Dr. J. Leon Williams, in which he claims to carry out nearly the same treatment as I do, but with this difference, that he does it for the *softened* dentine.

The priority always belonging to the person having made known through the scientific press a process of some kind, I must give precedence to Dr. J. L. Williams. I do not know in what year he began his researches on this subject, but, as I stated, mine began in February, 1895, the time when I first used hydronaphtol dissolved in alcohol and xylol. It is only gradually and by feeling my way that I arrived at the actual method that I am about to speak of. In any case, one point remains assured, I not only seek to sterilize softened dentine, but also and above all dentine seemingly healthy, and so I continue to work on the subject I have chosen, that is to say, to prevent the continuation of decay under good fillings. Besides, and above all, I am the first to have proved in an indisputable manner, by means of a very precise chemical reaction, (1) the penetration of the antiseptic into the dentine; (2) the complete sterilization of the latter.

It often happens that two authors come to the same results at the same epoch, without knowing each other. This was what happened in the case of Pasteur and Sternberg concerning the discovery of the pneumococcus. Pasteur published his results in the *Bulletin de l'Académie des Sciences* in January, 1881, whereas Sternberg, who had arrived at the same results without knowing of the experiments of Pasteur, published his, in the same year, in the April number of the *American Journal of Medical Science.*

Does it follow that Sternberg's work was of no value? Certainly not, since the results obtained were exactly the same. It is therefore the same thing in my opinion for the subject we are speaking of, with the only difference that, if Dr. J. Leon Williams was the first to publish in an interview his process of treatment of *softened* dentine for its sterilization, I am the first to have proved the complete sterilization, not only of softened dentine, but of the dentine covering the pulp, and containing always, notwithstanding its healthy look, great quantities of micro-organisms, showing themselves in course of time.

With this understanding, let us see again how we are to operate in order to obtain a perfect sterilization. (1) Mechanical cleansing of the cavity; (2) dehydration, not with very hot air, but cool air, to which is added afterwards the energetic action of alcohol of different strengths; (3) dry, with hot air, and substituting a mixture of alcohol, xylol, geranium essence, and hydronaphtol for the alcohol alone. We have two ways: either the filling may be done in the same sitting, or it will only be done twenty-four or forty-eight hours after.

CASE I.—The filling is done immediately when we have a rather small cavity, in which it is not possible to fix a temporary stopping.

CASE II.—The filling is accomplished only after twenty-four hours' time, during which a dressing of the mixture above described is left in the cavity, after having covered it with gutta-percha or wax. We can with great advantage make a paste with hydronaphtol and put it in the bottom of the cavity, in which it is retained by the dressing moistened with the liquid, and this covered by gutta-percha. There is a point to which we must draw special attention. When once the manipulations are begun, the saliva must be prevented from coming in contact with the cavity. Having seen the results obtained by experiments on the blocks of ivory I have already described, I wished to see what would happen in living teeth *in situ*. For this purpose I extracted two teeth, after having placed for five minutes in their cavities the same dressing used for the ivory blocks. One of these, a first right upper bicuspid affected with pulpitis, was submitted to dehydration by heat and alcohol of different strengths; it was soaked through. The other, a right upper wisdom-tooth growing outward, was treated only by burring. The dressing remained the same time as for the bicuspid; the staining reagent, however, did not soak in, but remained exclusively on the sides of the cavity. The experiment is therefore conclusive. When once I was sure that the penetration would be effected in the whole of the organ, I sought to see if the antiseptic employed also penetrated. I made a first experiment on blocks of ivory prepared in the same way as the other two, but with the difference that hydronaphtol was used instead of methylene-blue. The liquid was placed in the cavity and covered with a pad of wax during twenty-four hours. After that time, the cylinder was cut longitudinally, freed from the liquid, dried in the incubator, and examined with regard to the chemical reaction. The reagent employed is acid nitrate of mercury, which possesses the property, when placed upon a section of dentine, of staining it in a manner differing according to the presence or absence of hydronaphtol.

In the first case the reaction obtained is a dark yellow, whereas in the second it is pink.

I wished to see on a tooth that was to be extracted what would be produced. Here is a right upper lateral incisor having occasioned a great many abscesses, and moreover suffering from pyorrhœa. I entirely emptied the pulp-chamber, and after having operated as described, I left, during twenty-four hours, a wadding of the hydronaphtolized liquid, covered with a gutta-percha stopping. Acid nitrate of mercury shows us that the whole of the root is soaked through and especially the apical portions.

We had still to prove that this hydronaphtol acted efficaciously as an antiseptic on the micro-organisms contained in the dentine, whether healthy or softened.

Two sorts of experiments have been made on this subject: (1) Decayed teeth occasioning a defective dental arch have been extracted after having been submitted to the treatment as described during twenty-four hours, some of them having been submitted to the treatment indicated, and the others after a slight and simple dehydration with the aid of warm air. (2) Experiments have been made on living teeth treated in this manner, and which have been stopped for one, two, and three years, and in which I had been obliged to leave a thin layer of yellow dentine over the pulp.

Case I.—Left upper first bicuspid. Decay in the second degree, mesial surface; the cavity is cleaned as thoroughly as possible; nevertheless there remains a portion of yellow dentine. I dry, after using alcohol at seventy-five per cent., eighty per cent., and ninety per cent., and absolute as a dressing during twenty-four hours, and stopping with gutta-percha. Extraction: the tooth is passed in the flame of alcohol without having taken off the gutta-percha, is enveloped in sterilized paper, and broken in a vice. The pieces sown in the different nutrient media, both solid and liquid, in the air and in the vacuum, did not grow at all. Right upper wisdom-tooth; decay of the crown. Mechanical cleaning and simple drying with warm air. No alcohol used; same dressing and manual treatment as before. Development in every nutrient medium, and much more rapidly in the liquids.

Case II.—(*a*) The rubber dam having been placed on the first right upper molar filled with cement in 1898 on the mesial side, the cement is taken out with burs. When the cavity is quite empty I use round burs, previously passed in the flame of an alcohol lamp, dust of dentine being still yellow, but quite hard. I inoculate this dust in different media; nothing developed.

(*b*) Right upper central incisor, distal side, filled in July, 1899. Same operation; same negative result as to development.

(*c*) Right lower first molar, filled on the grinding surface in January, 1900. Same pro-manipulation; same negative results.

From all these observations it results that dental decay may be easily stopped by employing precise methods, and that we have no longer to fear at present the continuation of the destructive progress under good fillings.—*Journal of British Dental Association.*

